# 2-Phenyl-1*H*-1,3,7,8-tetra­azacyclo­penta­[*l*]phenanthrene

**DOI:** 10.1107/S1600536809017498

**Published:** 2009-05-20

**Authors:** Dong-Ming Liu, Xiu-Ying Li, Xiang-Cheng Wang, Chun-Xiang Li, Chun-Bo Liu

**Affiliations:** a Affiliated Hospital, Jiangsu University, Zhenjiang 212001, People’s Republic of China; b School of Chemistry and Chemical Engineering, Jiangsu University, Zhenjiang 212013, People’s Republic of China

## Abstract

There are two mol­ecules in the asymmetric unit of the title compound, C_19_H_12_N_4_, with dihedral angles of 2.41 (10) and 10.53 (12)° between the fused ring system and the pendant phenyl ring. In the crystal, mol­ecules are linked into chains by N—H⋯N hydrogen bonds and aromatic π–π stacking inter­actions [shortest centroid–centroid distance = 3.6176 (16) Å] complete the structure.

## Related literature

For the synthesis, see: Steck & Day (1943 ▶); For related structures, see: Che *et al.* (2008 ▶); Stephenson & Hardie (2006 ▶); Xi (2008 ▶).
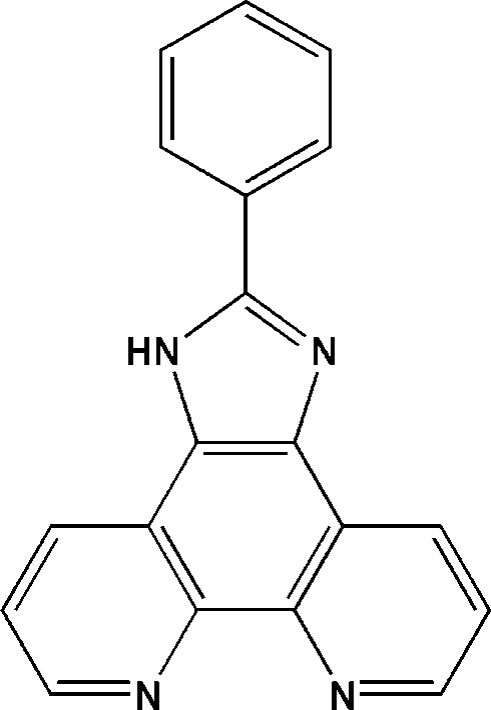


## Experimental

### 

#### Crystal data

C_19_H_12_N_4_
*M* *_r_* = 296.33
Triclinic, 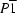

*a* = 10.016 (2) Å
*b* = 12.210 (2) Å
*c* = 12.415 (3) Å
α = 89.90 (3)°
β = 78.44 (3)°
γ = 77.96 (3)°
*V* = 1453.7 (5) Å^3^
*Z* = 4
Mo *K*α radiation
μ = 0.08 mm^−1^
*T* = 292 K
0.30 × 0.25 × 0.20 mm

#### Data collection

Rigaku R-AXIS RAPID diffractometer
Absorption correction: multi-scan (*ABSCOR*; Higashi, 1995 ▶) *T*_min_ = 0.975, *T*_max_ = 0.984
14453 measured reflections
6587 independent reflections
3657 reflections with *I* > 2σ(*I*)
*R*_int_ = 0.048

#### Refinement

*R*[*F* ^2^ > 2σ(*F* ^2^)] = 0.059
*wR*(*F* ^2^) = 0.156
*S* = 1.01
6587 reflections
415 parameters
H-atom parameters constrained
Δρ_max_ = 0.41 e Å^−3^
Δρ_min_ = −0.20 e Å^−3^

### 

Data collection: *PROCESS-AUTO* (Rigaku, 1998 ▶); cell refinement: *PROCESS-AUTO*; data reduction: *PROCESS-AUTO*; program(s) used to solve structure: *SHELXS97* (Sheldrick, 2008 ▶); program(s) used to refine structure: *SHELXL97* (Sheldrick, 2008 ▶); molecular graphics: *SHELXTL* (Sheldrick, 2008 ▶); software used to prepare material for publication: *SHELXL97*.

## Supplementary Material

Crystal structure: contains datablocks global, I. DOI: 10.1107/S1600536809017498/hb2971sup1.cif

Structure factors: contains datablocks I. DOI: 10.1107/S1600536809017498/hb2971Isup2.hkl

Additional supplementary materials:  crystallographic information; 3D view; checkCIF report

## Figures and Tables

**Table 1:** Hydrogen-bond geometry (Å, °)

*D*—H⋯*A*	*D*—H	H⋯*A*	*D*⋯*A*	*D*—H⋯*A*
N3—H3⋯N5^i^	0.86	2.10	2.932 (3)	164
N7—H7⋯N1	0.86	2.12	2.951 (2)	163

Symmetry code: (i) 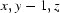.

**Fig. 1.**
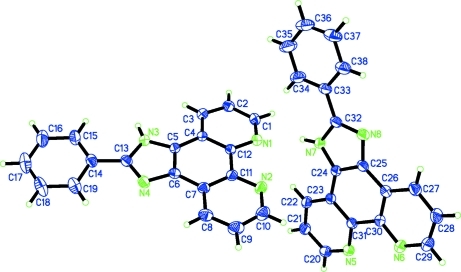
The structure of (I). Displacement ellipsoids are drawn at the 30% probability level (arbitrary spheres for the H atoms).

**Fig. 2.**
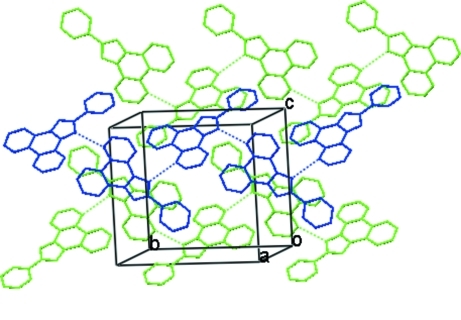
A view of the crystal packing, showing the N—H···N hydrogen bonds and π-π stacking interactions. H atoms have been omitted.

## supplementary crystallographic information

### Comment

1,10-Phenanthroline (phen) or its derivatives, as an important chelating ligands with excellent coordinating abilities and fruitful aromatic systems, have been extensively used to build supramolecular architectures (Che, Liu *et al.*, 2008; Stephenson, Hardie *et al.*, 2006). We report here the synthesis and structure of the title compound, namely, C_19_H_12_N_4_ (I), using the phen derivative 2-Phenyl-1*H*-1,3,7,8-tetraazacyclopenta- [*l*]phenanthrene (*L*).

The asymmetric unit of (I) consists of two independent *L* molecules (Fig.1). The two phenyl rings are slightly twisted with respect to the fused-ring system [dihedral angles = 1.34 and 1.54 °], which is different from a related compound that has been reported (Xi, 2008). In the crystal structure, N—H···N hydrogen bonds (Table 1) link the molecules into chains along the *b* axis. The neighbouring chains interact through π-π contact between two *L* ligands [centroid separation = 3.541 Å], leading to the ultimate supramolecular structure (Fig. 2).

### Experimental

The *L* ligand was synthesized according to the literature method of Steck & Day (1943): a mixture of *L*, MnCl_2_ and water in a molar ratio of 2:1:5000 was sealed in a Teflon-lined autoclave and heated to 413 K for 3 d. Upon cooling and opening the bomb, accidentally, pale yellow blocks of (I) were obtained.

### Refinement

The H atoms were positioned geometrically (C—H = 0.93 Å, N—H = 0.86Å) and refined as riding, with *U*_iso_(H) = 1.2*U*_eq_(carrier).

### Crystal data

**Table:**

C_19_H_12_N_4_	*Z* = 4
*M**_r_* = 296.33	*F*(000) = 616
Triclinic, *P*1	*D*_x_ = 1.354 Mg m^−^^3^
Hall symbol: -P 1	Mo *K*α radiation, λ = 0.71073 Å
*a* = 10.016 (2) Å	Cell parameters from 3449 reflections
*b* = 12.210 (2) Å	θ = 3.0–27.5°
*c* = 12.415 (3) Å	µ = 0.08 mm^−^^1^
α = 89.90 (3)°	*T* = 292 K
β = 78.44 (3)°	Block, pale yellow
γ = 77.96 (3)°	0.30 × 0.25 × 0.20 mm
*V* = 1453.7 (5) Å^3^	

### Data collection

**Table:**

Rigaku R-AXIS RAPID diffractometer	6587 independent reflections
Radiation source: fine-focus sealed tube	3657 reflections with *I* > 2σ(*I*)
graphite	*R*_int_ = 0.048
Detector resolution: 10.0 pixels mm^-1^	θ_max_ = 27.5°, θ_min_ = 3.0°
ω scans	*h* = −12→12
Absorption correction: multi-scan (*ABSCOR*; Higashi, 1995)	*k* = −15→15
*T*_min_ = 0.975, *T*_max_ = 0.984	*l* = −16→16
14453 measured reflections	

### Refinement

**Table:**

Refinement on *F*^2^	Primary atom site location: structure-invariant direct methods
Least-squares matrix: full	Secondary atom site location: difference Fourier map
*R*[*F*^2^ > 2σ(*F*^2^)] = 0.059	Hydrogen site location: inferred from neighbouring sites
*wR*(*F*^2^) = 0.156	H-atom parameters constrained
*S* = 1.01	*w* = 1/[σ^2^(*F*_o_^2^) + (0.0719*P*)^2^ + 0.0624*P*] where *P* = (*F*_o_^2^ + 2*F*_c_^2^)/3
6587 reflections	(Δ/σ)_max_ < 0.001
415 parameters	Δρ_max_ = 0.41 e Å^−^^3^
0 restraints	Δρ_min_ = −0.20 e Å^−^^3^

### Special details

**Table:**

Geometry. All e.s.d.'s (except the e.s.d. in the dihedral angle between two l.s. planes) are estimated using the full covariance matrix. The cell e.s.d.'s are taken into account individually in the estimation of e.s.d.'s in distances, angles and torsion angles; correlations between e.s.d.'s in cell parameters are only used when they are defined by crystal symmetry. An approximate (isotropic) treatment of cell e.s.d.'s is used for estimating e.s.d.'s involving l.s. planes.
Refinement. Refinement of *F*^2^ against ALL reflections. The weighted *R*-factor *wR* and goodness of fit *S* are based on *F*^2^, conventional *R*-factors *R* are based on *F*, with *F* set to zero for negative *F*^2^. The threshold expression of *F*^2^ > σ(*F*^2^) is used only for calculating *R*-factors(gt) *etc*. and is not relevant to the choice of reflections for refinement. *R*-factors based on *F*^2^ are statistically about twice as large as those based on *F*, and *R*- factors based on ALL data will be even larger.

### Fractional atomic coordinates and isotropic or equivalent isotropic displacement parameters (Å^2^)

**Table:**

	*x*	*y*	*z*	*U*_iso_*/*U*_eq_	
C1	−0.0622 (2)	0.23344 (19)	0.81330 (18)	0.0490 (6)	
H1	−0.1221	0.2801	0.8697	0.059*	
C2	−0.0895 (2)	0.12895 (19)	0.79496 (18)	0.0484 (6)	
H2	−0.1658	0.1068	0.8380	0.058*	
C3	−0.0027 (2)	0.05936 (18)	0.71295 (17)	0.0450 (5)	
H3A	−0.0182	−0.0115	0.7001	0.054*	
C4	0.1104 (2)	0.09578 (17)	0.64798 (15)	0.0372 (5)	
C5	0.2049 (2)	0.03323 (16)	0.55681 (16)	0.0389 (5)	
C6	0.3066 (2)	0.07524 (17)	0.48872 (16)	0.0399 (5)	
C7	0.3291 (2)	0.18394 (18)	0.51052 (16)	0.0411 (5)	
C8	0.4316 (2)	0.2316 (2)	0.44514 (19)	0.0511 (6)	
H8	0.4916	0.1918	0.3843	0.061*	
C9	0.4418 (3)	0.3372 (2)	0.4723 (2)	0.0620 (7)	
H9	0.5088	0.3707	0.4300	0.074*	
C10	0.3510 (3)	0.3944 (2)	0.5639 (2)	0.0602 (7)	
H10	0.3590	0.4665	0.5808	0.072*	
C11	0.2416 (2)	0.24813 (17)	0.60234 (16)	0.0396 (5)	
C12	0.1303 (2)	0.20394 (17)	0.67106 (15)	0.0376 (5)	
C13	0.3167 (2)	−0.08769 (19)	0.42188 (16)	0.0441 (5)	
C14	0.3576 (2)	−0.1902 (2)	0.35036 (18)	0.0471 (6)	
C15	0.3037 (3)	−0.2845 (2)	0.3759 (2)	0.0621 (7)	
H15	0.2408	−0.2852	0.4419	0.075*	
C16	0.3416 (3)	−0.3775 (2)	0.3051 (2)	0.0745 (8)	
H16	0.3036	−0.4401	0.3231	0.089*	
C17	0.4350 (3)	−0.3777 (3)	0.2086 (3)	0.0802 (10)	
H17	0.4613	−0.4406	0.1609	0.096*	
C18	0.4895 (3)	−0.2857 (3)	0.1823 (2)	0.0781 (9)	
H18	0.5529	−0.2861	0.1163	0.094*	
C19	0.4521 (3)	−0.1917 (2)	0.2523 (2)	0.0631 (7)	
H19	0.4904	−0.1295	0.2335	0.076*	
C20	−0.0061 (2)	0.71983 (19)	0.57979 (18)	0.0495 (6)	
H20	−0.0384	0.7605	0.5233	0.059*	
C21	−0.0429 (2)	0.61739 (19)	0.60084 (18)	0.0485 (6)	
H21	−0.0989	0.5910	0.5600	0.058*	
C22	0.0046 (2)	0.55589 (18)	0.68273 (17)	0.0455 (5)	
H22	−0.0169	0.4862	0.6976	0.055*	
C23	0.0864 (2)	0.59938 (17)	0.74404 (16)	0.0382 (5)	
C24	0.1393 (2)	0.54495 (17)	0.83266 (16)	0.0379 (5)	
C25	0.2102 (2)	0.59356 (18)	0.89767 (16)	0.0406 (5)	
C26	0.2425 (2)	0.70078 (18)	0.87370 (16)	0.0405 (5)	
C27	0.3175 (2)	0.7535 (2)	0.93361 (18)	0.0510 (6)	
H27	0.3498	0.7192	0.9931	0.061*	
C28	0.3424 (3)	0.8564 (2)	0.9033 (2)	0.0599 (7)	
H28	0.3910	0.8936	0.9423	0.072*	
C29	0.2938 (3)	0.9040 (2)	0.8131 (2)	0.0611 (7)	
H29	0.3119	0.9737	0.7931	0.073*	
C30	0.1977 (2)	0.75555 (17)	0.78341 (16)	0.0404 (5)	
C31	0.1167 (2)	0.70490 (17)	0.71880 (16)	0.0384 (5)	
C32	0.1928 (2)	0.43446 (18)	0.96433 (16)	0.0415 (5)	
C33	0.2076 (2)	0.33528 (19)	1.03149 (17)	0.0447 (5)	
C34	0.1535 (3)	0.2436 (2)	1.0129 (2)	0.0654 (7)	
H34	0.1045	0.2443	0.9566	0.078*	
C35	0.1709 (3)	0.1517 (3)	1.0762 (2)	0.0797 (9)	
H35	0.1332	0.0908	1.0627	0.096*	
C36	0.2433 (3)	0.1487 (3)	1.1591 (2)	0.0751 (8)	
H36	0.2558	0.0859	1.2015	0.090*	
C37	0.2964 (3)	0.2383 (3)	1.1787 (2)	0.0709 (8)	
H37	0.3447	0.2371	1.2355	0.085*	
C38	0.2797 (3)	0.3312 (2)	1.11548 (19)	0.0603 (7)	
H38	0.3174	0.3918	1.1297	0.072*	
N1	0.04442 (19)	0.27076 (14)	0.75517 (14)	0.0444 (5)	
N2	0.2540 (2)	0.35239 (16)	0.62830 (15)	0.0488 (5)	
N3	0.21246 (18)	−0.07178 (14)	0.51377 (13)	0.0429 (4)	
H3	0.1617	−0.1184	0.5395	0.052*	
N4	0.37586 (19)	0.00010 (16)	0.40376 (14)	0.0461 (5)	
N5	0.0722 (2)	0.76364 (15)	0.63503 (14)	0.0460 (5)	
N6	0.2236 (2)	0.85701 (16)	0.75407 (15)	0.0505 (5)	
N7	0.12911 (18)	0.44362 (14)	0.87635 (13)	0.0403 (4)	
H7	0.0899	0.3947	0.8529	0.048*	
N8	0.24317 (19)	0.52409 (15)	0.98030 (14)	0.0441 (4)	

### Atomic displacement parameters (Å^2^)

**Table:**

	*U*^11^	*U*^22^	*U*^33^	*U*^12^	*U*^13^	*U*^23^
C1	0.0537 (14)	0.0477 (14)	0.0407 (12)	−0.0078 (11)	−0.0015 (11)	−0.0058 (11)
C2	0.0496 (13)	0.0490 (14)	0.0437 (12)	−0.0115 (11)	−0.0017 (11)	0.0028 (11)
C3	0.0518 (13)	0.0392 (12)	0.0451 (12)	−0.0118 (10)	−0.0098 (11)	0.0036 (10)
C4	0.0449 (12)	0.0340 (11)	0.0327 (10)	−0.0070 (9)	−0.0098 (9)	0.0033 (9)
C5	0.0489 (12)	0.0327 (11)	0.0346 (11)	−0.0057 (10)	−0.0103 (10)	0.0025 (9)
C6	0.0452 (12)	0.0406 (12)	0.0347 (11)	−0.0076 (10)	−0.0118 (10)	0.0055 (9)
C7	0.0446 (12)	0.0435 (13)	0.0382 (11)	−0.0108 (10)	−0.0140 (10)	0.0070 (10)
C8	0.0479 (14)	0.0620 (16)	0.0468 (13)	−0.0183 (12)	−0.0106 (11)	0.0090 (11)
C9	0.0588 (16)	0.0709 (18)	0.0647 (16)	−0.0337 (14)	−0.0118 (13)	0.0143 (14)
C10	0.0650 (17)	0.0540 (16)	0.0692 (17)	−0.0279 (13)	−0.0156 (14)	0.0053 (13)
C11	0.0452 (12)	0.0385 (12)	0.0385 (11)	−0.0092 (10)	−0.0162 (10)	0.0054 (9)
C12	0.0446 (12)	0.0364 (11)	0.0328 (10)	−0.0066 (9)	−0.0121 (9)	0.0031 (9)
C13	0.0481 (13)	0.0463 (13)	0.0351 (11)	−0.0030 (11)	−0.0094 (10)	0.0002 (10)
C14	0.0477 (13)	0.0498 (14)	0.0415 (12)	−0.0009 (11)	−0.0139 (10)	−0.0055 (11)
C15	0.0723 (17)	0.0529 (16)	0.0540 (15)	−0.0022 (13)	−0.0077 (13)	−0.0143 (13)
C16	0.086 (2)	0.0588 (18)	0.0757 (19)	−0.0058 (15)	−0.0186 (17)	−0.0197 (15)
C17	0.0701 (19)	0.081 (2)	0.082 (2)	0.0056 (17)	−0.0209 (17)	−0.0417 (18)
C18	0.0578 (17)	0.103 (3)	0.0629 (17)	−0.0055 (17)	0.0007 (14)	−0.0354 (18)
C19	0.0541 (15)	0.0762 (18)	0.0535 (15)	−0.0076 (13)	−0.0047 (12)	−0.0152 (14)
C20	0.0639 (15)	0.0469 (14)	0.0419 (12)	−0.0100 (12)	−0.0224 (11)	0.0082 (10)
C21	0.0564 (14)	0.0453 (14)	0.0483 (13)	−0.0087 (11)	−0.0235 (11)	0.0018 (11)
C22	0.0580 (14)	0.0366 (12)	0.0465 (12)	−0.0103 (11)	−0.0209 (11)	0.0018 (10)
C23	0.0429 (12)	0.0340 (12)	0.0366 (11)	−0.0050 (9)	−0.0092 (9)	−0.0008 (9)
C24	0.0468 (12)	0.0322 (11)	0.0343 (10)	−0.0053 (9)	−0.0103 (9)	0.0005 (9)
C25	0.0449 (12)	0.0423 (12)	0.0330 (11)	−0.0043 (10)	−0.0094 (9)	0.0003 (9)
C26	0.0414 (12)	0.0431 (13)	0.0361 (11)	−0.0079 (10)	−0.0069 (9)	−0.0017 (9)
C27	0.0543 (14)	0.0593 (16)	0.0434 (13)	−0.0175 (12)	−0.0142 (11)	−0.0027 (11)
C28	0.0663 (16)	0.0649 (17)	0.0567 (15)	−0.0290 (14)	−0.0160 (13)	−0.0051 (13)
C29	0.0739 (18)	0.0540 (16)	0.0648 (16)	−0.0317 (14)	−0.0175 (14)	0.0048 (13)
C30	0.0456 (12)	0.0366 (12)	0.0393 (11)	−0.0104 (10)	−0.0073 (10)	−0.0009 (9)
C31	0.0434 (12)	0.0368 (12)	0.0337 (11)	−0.0052 (9)	−0.0087 (9)	−0.0003 (9)
C32	0.0459 (13)	0.0442 (13)	0.0321 (10)	−0.0027 (10)	−0.0095 (9)	0.0022 (9)
C33	0.0474 (13)	0.0467 (13)	0.0350 (11)	−0.0013 (10)	−0.0059 (10)	0.0067 (10)
C34	0.085 (2)	0.0583 (17)	0.0612 (16)	−0.0206 (15)	−0.0298 (14)	0.0237 (13)
C35	0.103 (2)	0.0656 (19)	0.079 (2)	−0.0267 (17)	−0.0296 (18)	0.0329 (16)
C36	0.080 (2)	0.070 (2)	0.0656 (18)	−0.0016 (16)	−0.0087 (16)	0.0328 (15)
C37	0.0715 (19)	0.088 (2)	0.0504 (15)	−0.0020 (17)	−0.0226 (14)	0.0220 (15)
C38	0.0646 (17)	0.0678 (18)	0.0473 (14)	−0.0062 (14)	−0.0167 (12)	0.0108 (13)
N1	0.0540 (11)	0.0385 (10)	0.0400 (10)	−0.0086 (9)	−0.0094 (9)	−0.0009 (8)
N2	0.0573 (12)	0.0411 (11)	0.0536 (11)	−0.0171 (9)	−0.0179 (9)	0.0032 (9)
N3	0.0521 (11)	0.0370 (10)	0.0377 (10)	−0.0091 (8)	−0.0049 (9)	−0.0007 (8)
N4	0.0486 (11)	0.0494 (12)	0.0369 (9)	−0.0057 (9)	−0.0052 (8)	−0.0018 (9)
N5	0.0581 (12)	0.0423 (11)	0.0408 (10)	−0.0114 (9)	−0.0165 (9)	0.0046 (8)
N6	0.0622 (13)	0.0431 (11)	0.0516 (11)	−0.0204 (10)	−0.0146 (10)	0.0027 (9)
N7	0.0514 (11)	0.0349 (10)	0.0373 (9)	−0.0093 (8)	−0.0149 (8)	0.0042 (8)
N8	0.0496 (11)	0.0466 (11)	0.0369 (9)	−0.0079 (9)	−0.0134 (8)	0.0042 (8)

### Geometric parameters (Å, °)

**Table:**

C1—N1	1.327 (3)	C20—C21	1.386 (3)
C1—C2	1.388 (3)	C20—H20	0.9300
C1—H1	0.9300	C21—C22	1.366 (3)
C2—C3	1.362 (3)	C21—H21	0.9300
C2—H2	0.9300	C22—C23	1.402 (3)
C3—C4	1.405 (3)	C22—H22	0.9300
C3—H3A	0.9300	C23—C31	1.405 (3)
C4—C12	1.414 (3)	C23—C24	1.420 (3)
C4—C5	1.426 (3)	C24—N7	1.365 (3)
C5—N3	1.371 (2)	C24—C25	1.384 (3)
C5—C6	1.374 (3)	C25—N8	1.377 (3)
C6—N4	1.371 (3)	C25—C26	1.430 (3)
C6—C7	1.427 (3)	C26—C27	1.399 (3)
C7—C8	1.402 (3)	C26—C30	1.407 (3)
C7—C11	1.414 (3)	C27—C28	1.369 (3)
C8—C9	1.363 (3)	C27—H27	0.9300
C8—H8	0.9300	C28—C29	1.388 (4)
C9—C10	1.390 (3)	C28—H28	0.9300
C9—H9	0.9300	C29—N6	1.319 (3)
C10—N2	1.320 (3)	C29—H29	0.9300
C10—H10	0.9300	C30—N6	1.352 (3)
C11—N2	1.350 (3)	C30—C31	1.468 (3)
C11—C12	1.459 (3)	C31—N5	1.358 (3)
C12—N1	1.359 (3)	C32—N8	1.327 (3)
C13—N4	1.327 (3)	C32—N7	1.365 (3)
C13—N3	1.367 (3)	C32—C33	1.468 (3)
C13—C14	1.472 (3)	C33—C38	1.377 (3)
C14—C19	1.381 (3)	C33—C34	1.380 (3)
C14—C15	1.381 (3)	C34—C35	1.371 (4)
C15—C16	1.377 (3)	C34—H34	0.9300
C15—H15	0.9300	C35—C36	1.370 (4)
C16—C17	1.364 (4)	C35—H35	0.9300
C16—H16	0.9300	C36—C37	1.354 (4)
C17—C18	1.360 (4)	C36—H36	0.9300
C17—H17	0.9300	C37—C38	1.380 (4)
C18—C19	1.381 (3)	C37—H37	0.9300
C18—H18	0.9300	C38—H38	0.9300
C19—H19	0.9300	N3—H3	0.8600
C20—N5	1.328 (3)	N7—H7	0.8600
			
N1—C1—C2	123.9 (2)	C21—C22—C23	119.0 (2)
N1—C1—H1	118.1	C21—C22—H22	120.5
C2—C1—H1	118.1	C23—C22—H22	120.5
C3—C2—C1	118.9 (2)	C22—C23—C31	118.7 (2)
C3—C2—H2	120.5	C22—C23—C24	124.4 (2)
C1—C2—H2	120.5	C31—C23—C24	116.91 (18)
C2—C3—C4	119.3 (2)	N7—C24—C25	105.51 (18)
C2—C3—H3A	120.3	N7—C24—C23	131.11 (18)
C4—C3—H3A	120.3	C25—C24—C23	123.3 (2)
C3—C4—C12	118.36 (18)	N8—C25—C24	110.69 (19)
C3—C4—C5	124.80 (19)	N8—C25—C26	128.53 (19)
C12—C4—C5	116.80 (18)	C24—C25—C26	120.8 (2)
N3—C5—C6	105.81 (17)	C27—C26—C30	118.2 (2)
N3—C5—C4	130.73 (19)	C27—C26—C25	124.0 (2)
C6—C5—C4	123.36 (19)	C30—C26—C25	117.79 (19)
N4—C6—C5	110.98 (18)	C28—C27—C26	119.0 (2)
N4—C6—C7	128.25 (19)	C28—C27—H27	120.5
C5—C6—C7	120.76 (18)	C26—C27—H27	120.5
C8—C7—C11	118.0 (2)	C27—C28—C29	118.6 (2)
C8—C7—C6	123.79 (19)	C27—C28—H28	120.7
C11—C7—C6	118.21 (19)	C29—C28—H28	120.7
C9—C8—C7	118.9 (2)	N6—C29—C28	124.3 (2)
C9—C8—H8	120.6	N6—C29—H29	117.8
C7—C8—H8	120.6	C28—C29—H29	117.8
C8—C9—C10	119.2 (2)	N6—C30—C26	122.15 (19)
C8—C9—H9	120.4	N6—C30—C31	117.3 (2)
C10—C9—H9	120.4	C26—C30—C31	120.52 (19)
N2—C10—C9	124.0 (2)	N5—C31—C23	121.66 (18)
N2—C10—H10	118.0	N5—C31—C30	117.79 (19)
C9—C10—H10	118.0	C23—C31—C30	120.55 (19)
N2—C11—C7	122.30 (19)	N8—C32—N7	112.31 (19)
N2—C11—C12	117.46 (19)	N8—C32—C33	123.9 (2)
C7—C11—C12	120.21 (18)	N7—C32—C33	123.74 (19)
N1—C12—C4	121.24 (19)	C38—C33—C34	117.9 (2)
N1—C12—C11	118.20 (18)	C38—C33—C32	119.7 (2)
C4—C12—C11	120.53 (18)	C34—C33—C32	122.4 (2)
N4—C13—N3	112.41 (17)	C35—C34—C33	120.9 (3)
N4—C13—C14	123.79 (19)	C35—C34—H34	119.5
N3—C13—C14	123.8 (2)	C33—C34—H34	119.5
C19—C14—C15	118.4 (2)	C36—C35—C34	120.5 (3)
C19—C14—C13	118.8 (2)	C36—C35—H35	119.7
C15—C14—C13	122.9 (2)	C34—C35—H35	119.7
C16—C15—C14	121.1 (3)	C37—C36—C35	119.2 (3)
C16—C15—H15	119.5	C37—C36—H36	120.4
C14—C15—H15	119.5	C35—C36—H36	120.4
C17—C16—C15	119.8 (3)	C36—C37—C38	120.8 (3)
C17—C16—H16	120.1	C36—C37—H37	119.6
C15—C16—H16	120.1	C38—C37—H37	119.6
C18—C17—C16	119.9 (2)	C33—C38—C37	120.7 (3)
C18—C17—H17	120.1	C33—C38—H38	119.7
C16—C17—H17	120.1	C37—C38—H38	119.7
C17—C18—C19	120.9 (3)	C1—N1—C12	118.24 (18)
C17—C18—H18	119.6	C10—N2—C11	117.7 (2)
C19—C18—H18	119.6	C13—N3—C5	106.42 (18)
C14—C19—C18	120.0 (3)	C13—N3—H3	126.8
C14—C19—H19	120.0	C5—N3—H3	126.8
C18—C19—H19	120.0	C13—N4—C6	104.37 (17)
N5—C20—C21	124.2 (2)	C20—N5—C31	117.64 (19)
N5—C20—H20	117.9	C29—N6—C30	117.6 (2)
C21—C20—H20	117.9	C24—N7—C32	107.11 (17)
C22—C21—C20	118.7 (2)	C24—N7—H7	126.4
C22—C21—H21	120.6	C32—N7—H7	126.4
C20—C21—H21	120.6	C32—N8—C25	104.37 (17)

### Hydrogen-bond geometry (Å, °)

**Table:**

*D*—H···*A*	*D*—H	H···*A*	*D*···*A*	*D*—H···*A*
N3—H3···N5^i^	0.86	2.10	2.932 (3)	164
N7—H7···N1	0.86	2.12	2.951 (2)	163

Symmetry codes: (i) *x*, *y*−1, *z*.
